# Prognostic impact of ER-staining patterns and heterogeneity of ER positive HER2 negative breast cancer

**DOI:** 10.1007/s12282-025-01716-4

**Published:** 2025-05-18

**Authors:** Yumiko Akita, Ravi Velaga, Madoka Iwase, Satoko Shimada, Toyone Kikumori, Dai Takeuchi, Yuko Takano, Takahiro Ichikawa, Tomoki Ebata, Norikazu Masuda

**Affiliations:** 1 Department of Breast and Endocrine Surgery, Nagoya University Graduate School of Medicine, 65 Tsurumai-cho, Showa-ku, Nagoya, Aichi 466-8550 Japan; 2 Department of Pathology, Nagoya University Hospital, 65 Tsurumai-cho, Showa-ku, Nagoya, Aichi 466-8550 Japan; 3 Department of Clinical Oncology and Chemotherapy, Nagoya University Hospital, 65 Tsurumai-cho, Showa-ku, Nagoya, Aichi 466-8550 Japan; 4 Division of Surgical Oncology, Department of Surgery, Nagoya University Graduate School of Medicine, 65 Tsurumai-cho, Showa-ku, Nagoya, Aichi 466-8550 Japan; 5 Department of Breast Surgery, Graduate School of Medicine, Kyoto University, 54 Shogoin Kawahara-cho, Sakyo-ku, Kyoto, 606-8507 Japan

**Keywords:** ER-positive breast cancer, ER-staining patterns, Heterogeneity, Tumor-infiltrating lymphocytes, Transcriptome

## Abstract

**Background:**

Estrogen receptor (ER) expression is critical in breast cancer treatment. While low ER (1–9%) resembles triple-negative cancer with chemotherapy efficacy, the significance of “intermediate expression” (≥ 10%) and the therapeutic efficacy remain unclear. This study explores the differences in staining patterns and molecular characteristics of ER-low to intermediate expression to guide treatment.

**Methods:**

A total of 104 breast cancer patients treated between January 2008 and July 2024 with an Allred Proportion Score (PS) of 2–4 were included. PS2 (*n* = 21) was classified as ER-low, while PS3 (*n* = 26) and PS4 (*n* = 57) as ER-intermediate (ER-int). ER-int was further divided by ER staining pattern: “Island” (heterogeneous) and “Scatter,” (uniform) subgroups. The prognosis, clinical factors, and gene expression profiles (*n* = 11) were analyzed.

**Results:**

The Island subgroup was associated with poorest prognosis (*p* = 0.0116), particularly among the patients treated with endocrine-only treatment patients (*p* < 0.0001). Elevated tumor-infiltrating lymphocyte (TIL) levels correlated with worse prognosis in endocrine-only treatment patients (*p* < 0.0043), with TIL levels highest in ER-low, followed by Island and Scatter subgroups. Island tumors were enriched in CD36, GZMB, and type I interferon genes; additionally, 23 “ISLAND” genes showed significant prognostic differences in the TCGA BRCA ER-int (10–69%) cohort.

**Conclusion:**

This study emphasizes the importance of recognizing heterogeneity within the ER-int subtype. Identifying distinct ER staining patterns and prognostic significance of TILs and transcriptome in ER-int tumors suggests the need for individualized treatment strategies for Island subtype.

**Supplementary Information:**

The online version contains supplementary material available at 10.1007/s12282-025-01716-4.

## Introduction

Estrogen receptor (ER)-positive breast cancer, accounting for 75% of all cases [1], relies on estrogen signaling for growth. The 2010 ASCO/CAP guidelines define ER-positive breast cancer as having ≥ 1% ER expression, with staining intensity varying from 1 to 100% [2]. The endocrine therapies have significantly improved survival in ER-positive patients [3, 4]. However, the efficacy of endocrine therapy varies depending on ER expression levels. ER-high expression (≥ 67%) demonstrates significantly greater sensitivity to endocrine therapy compared to lower ER levels [5]. Additionally lower ER expression potentially correlated with reduced endocrine therapy sensitivity [6, 7].

The 2020 ASCO/CAP guidelines introduced a new ‘ER-low positive’ category, defined as 1–9% ER expression [8]. The studies indicate that ER-low expression is associated with clinicopathological features and prognoses similar to those of triple negative (TN) breast cancer, and these patients may benefit from chemotherapy [9–12]. Recent research suggests potential benefits of immune checkpoint inhibitors in these patients [13]. Although ER-low is managed similarly to TN, the treatment strategy for those with an ER level above 10% but below ER-high, considered ER-intermediate (ER-int) are indicated for endocrine therapy in the same way as ER-high. However, there is no established view on whether they should receive the same treatment as patients with ER-high expression.

The exact significance of the ER-int population remains ambiguous. The molecular profiling has shown that patients with ER-low and ER-int expression exhibit heterogeneous [14–16]. Cancer heterogeneity has long been recognized through the clonal expansion of cancer cells [17], but advancements in next-generation sequencing have highlighted the role of intra-tumor heterogeneity within the tumor microenvironment (TME) and its impact on treatment responses [18–22]. Establishing the prognosis of the ER-int group as a potential guide for treatment is essential. This study aims to determine whether ER-int breast cancer is heterogeneous by examining the clinical and pathological features of ER-int groups, along with prognostic and transcriptomic differences.

## Materials and methods

### Sample inclusion in survival analysis

**Fig. 1:**
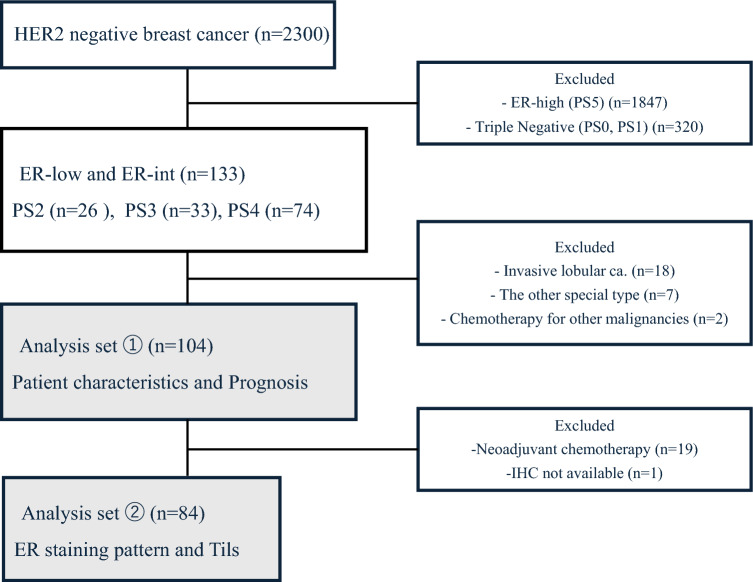
Flowchart of this study. There were 2300 HER2-negative breast cancer surgery patients during the relevant period. ER-high (PS5) was 1847 cases, and Triple Negative (PS0 and PS1) were 320 cases were excluded. 133 patients were classified as ER-low (PS2, *n* = 26) and ER-int(PS3 and PS4, *n* = 107). A total of 104 cases, excluding invasive lobular carcinoma (*n* = 18), special histological types (*n* = 7) and cases with chemotherapy for other malignancies (*n* = 2) were first analyzed for patient background and prognosis. Next, 84 surgical specimens were analyzed for ER staining patterns and TILs after excluding cases with neoadjuvant chemotherapy

This study was conducted with ethical approval from the Institutional Review Board of Nagoya University Hospital and informed consent was obtained from all patients. This study included consecutive patients with early-stage, potentially curative invasive breast cancer treated at Nagoya University Hospital from January 2008 to July 2024. ER expression was assessed using immunohistochemistry (IHC) and scored by proportion score (PS) according to the Allred scoring system. The intensity scores from the Allred scoring system were not included in the patient selection criteria, only the distribution was used as a selection criterion. A total of 2300 HER2-negative breast cancer patients were initially screened, of 2167 patients were excluded for the following reasons: 1847 patients with ER-high expression (PS5) and 320 patients with TN breast cancer (PS0, PS1). This left a cohort of 133 patients with ER-low and ER-int (PS2-PS4). PS2 (*n* = 23) was categorized as ER-low, whereas PS3 (*n* = 25) and PS4 (*n* = 56) were categorized as ER-int. Only cases diagnosed with invasive ductal carcinoma were included; the patients with invasive lobular carcinoma (*n* = 18) and other special histologic types (*n* = 7) were excluded. After excluding two patients who had received chemotherapy for other malignancies, 104 patients remained in the first analysis set. ER staining rates were initially assessed through surgical specimens in patients who had not received neoadjuvant chemotherapy. For patients who had received neoadjuvant chemotherapy, the classification was based on biopsy specimens. These 104 patients were classified as ER-low (*n* = 23) and ER-int (*n* = 81), and an analysis of disease-free survival (DFS) was performed (Fig. 1). DFS was defined as the time from the date of surgery to the date of first documented distant metastasis. Deaths from other causes were not considered as events and patients without distant metastasis were censored at the date of last follow-up.

Nineteen patients who had received neoadjuvant chemotherapy and one patient with no IHC result were excluded, leaving 84 cases as the second analysis set (Fig. 1).

## ER-int staining patterns: Island or Scatter

**Fig. 2:**
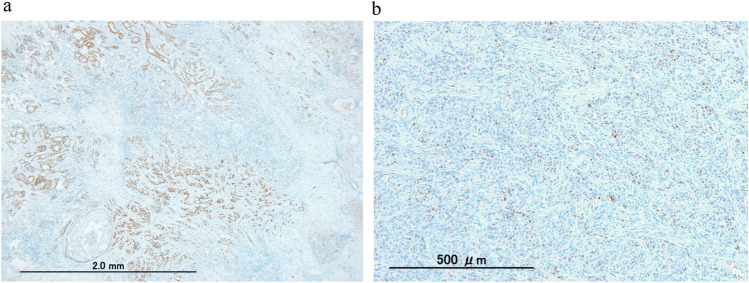
Staining pattern of two cases identified as PS3. **a** An example of the Island type. A significant portion of ER-positive cells is concentrated in some glands, with sparse distribution elsewhere, resulting in an overall heterogeneous distribution within the tumor. **b** An example of the Scatter type. ER-positive cells are scattered at nearly uniform proportions

Histological examination of ER-int samples identified two distinct staining patterns: “Island” and “Scatter”. Island patterns displayed clusters of ER-positive cells with marked heterogeneity, while Scatter patterns exhibited a uniform distribution of ER-positive cells across cancer nest (Fig. 2). Island and Scatter showed marked differences in the distribution of ER-positive cells, even with the same PS score. In the Island pattern, some cancer nests were rich in ER-positive cells, while others had only a few, creating areas that resembled ER-high expression and ER-low/TN-like regions within the same tumor. This resulted in greater intra-tumor heterogeneity compared to the Scatter pattern. To ensure objective classification, only cases with clear intra-tumoral heterogeneity were defined as Island, while cases with gradual transitions, subtle staining variations, or ambiguous heterogeneity were classified as Scatter. Pathologists and a trained clinician assessed ER staining patterns on IHC slides of the entire tumor in surgical specimens. The regions with suspected internal necrosis or staining variability due to poor fixation were excluded to ensure accurate Island pattern identification. The cases treated with neoadjuvant chemotherapy, which may alter ER staining patterns, were also excluded.

## Tumor infiltrating lymphocytes (TILs) analysis

For the determination of TILs, 84 patients were included, excluding those who had received neoadjuvant chemotherapy as well as staining pattern. Only surgical specimens from patients without neoadjuvant chemotherapy were used to assess TILs. Given the study’s focus on staining pattern heterogeneity, the entire tumor area was analyzed, excluding biopsy specimens to prevent sampling bias. TILs were evaluated on H&E-stained slides following the TILs Working Group guidelines (2014) [23], with at least one slide containing the maximum tumor diameter analyzed to determine the TIL average across the tumor. A cutoff value of 20% was used to stratify TIL levels. Although TILs are a continuous variable with no established universal cutoff [23], a 20% threshold was selected based on previous studies in breast cancer. Prior reports have used cutoff values ranging from 10 to 40% in triple-negative and HER2-positive breast cancers [24], and thresholds of 10–30% have been suggested even in hormone receptor-positive cases [24, 25]. In addition, 20% cutoff provided a relatively balanced distribution of cases in our study.

## Statistical analysis

For statistical analysis, we used JMP^®^ Pro 17 (SAS Institute Inc., Cary, NC, USA), which facilitated data organization, analysis, and graph creation. The categorical variables were analyzed via Fisher’s exact test, while nonparametric statistical tests, such as the Mann‒Whitney *U* test for two-group comparisons and the Kruskal‒Wallis test for comparisons across three or more groups, were used for continuous variables. A significance level of *α* = 0.05 was applied to all statistical tests.

## Transcriptomic profiling and functional analysis for ER-int Tumors

### Transcriptomic characterization of subtypes

Whole RNA sequencing of breast cancer subtypes was performed to characterize and understand the ER-int group in comparing with ER-high, low and TN tumor (*n* = 11). RNA was extracted from ER-int tumors (3 Island and 2 Scatter), ER-high (*n* = 2), ER-low (*n* = 2) and TN (*n* = 2). RNA was extracted from Formalin-Fixed, Paraffin-Embedded (FFPE) of surgical specimens with macrodissection of the tumor area. RNA was isolated using Qiagen RNA extraction kit and sequencing libraries were prepared according to the TruSeq RNA Exome protocol (Illumina). The libraries were pooled based on the TapeStation D1000 QC measurements and sequenced on an Illumina NovaSeq X Plus platform.

### RNA sequencing data processing and differentially expressed genes (DEGs) identification

Paired-end raw FASTQ files were uploaded to QIAGEN CLC-GWB 24.0.2 version (https://digitalinsights.qiagen.com/) to identify DEGs between the tumor subtypes. Normalized gene expression data were used to calculate principal components across different treatment groups. Venn analysis was employed to identify significant DEGs shared between tumor subtypes. Genes with log fold changes ≤ 1.5 or > 1.5 and *p*-values < 0.05 were deemed significant for downstream transcriptomic analysis, including gene set enrichment and functional annotation.

### Gene set enrichment analysis (GSEA) of Island and Scatter transcriptomes

GSEA v4.3.2 was used to compare gene enrichment between Island (study group) and Scatter (control group) samples [26]. Cancer hallmark enrichment analysis identified gene sets up-regulated (NES > 0) or down-regulated (NES < 0) in Island samples. Significantly enriched gene sets were defined by false discovery rate (FDR)-corrected *p*-values < 0.01.

### Functional annotation of DEGs

The Database for Annotation, Visualization, and Integrated Discovery (DAVID) Knowledgebase (v2023q4) [27, 28] was used to explore the biological significance of DEGs (*p* < 0.05, *n* = 1546) between Island and Scatter samples. Kyoto Encyclopedia of Genes and Genomes(KEGG) [29] pathway enrichment analysis identified functionally relevant DEGs in Island tumors, which we defined as the ISLANDER gene set (*n* = 23).

### ISLANDER gene set relevance in The Cancer Genome Atlas (TCGA) survival analysis

The TCGA BRCA dataset [30] was stratified by ER expression level (ER-high: 70–99%, ER-int: 10–69%, and ER-low: < 10%) using Xena [31] tool to identify ER-int subgroups. Kaplan–Meier analyses of overall survival (OS), disease-specific survival (DSS), disease-free interval (DFI), and progression-free interval (PFI) in the TCGA BRCA ER-int subgroup were conducted based on the median expression of the ISLANDER gene set (*n* = 23). Log-rank p-values and test statistics are presented in the respective figures, following TCGA-defined survival criteria [32].

## Results

### Clinical and prognostic comparison of ER-low and ER-int

**Fig. 3:**
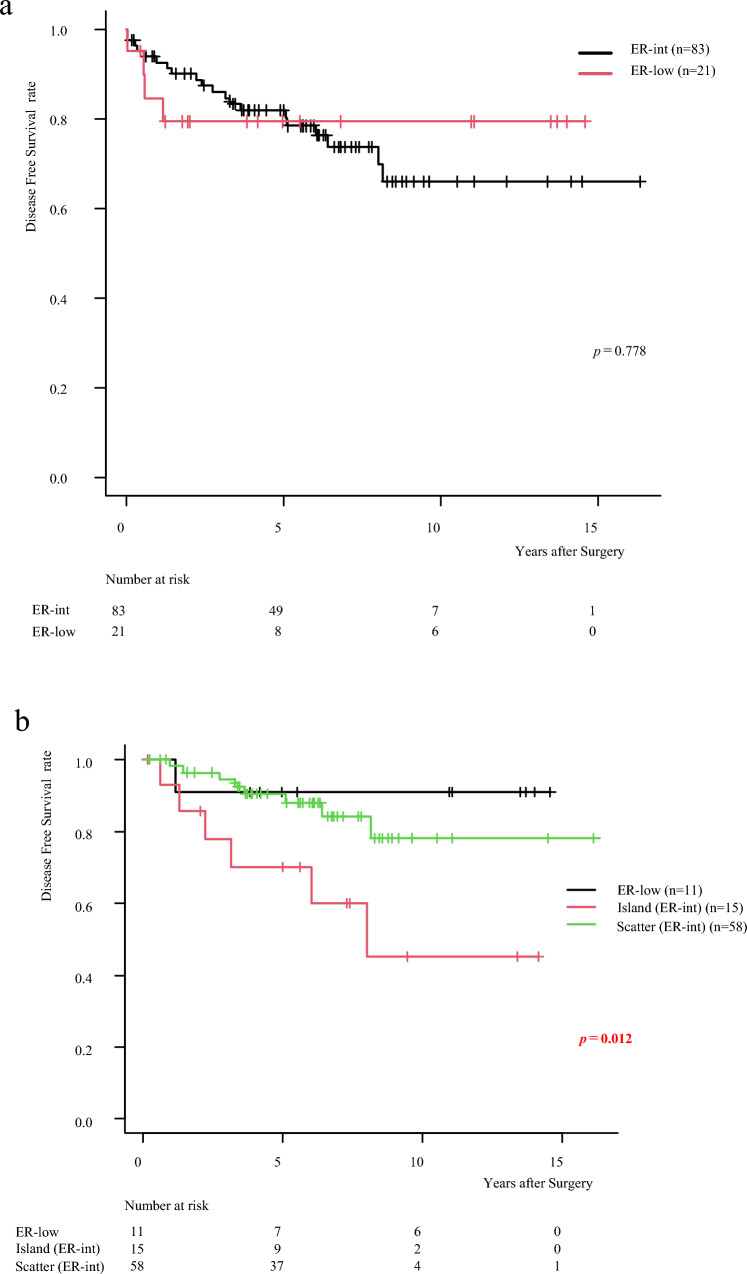

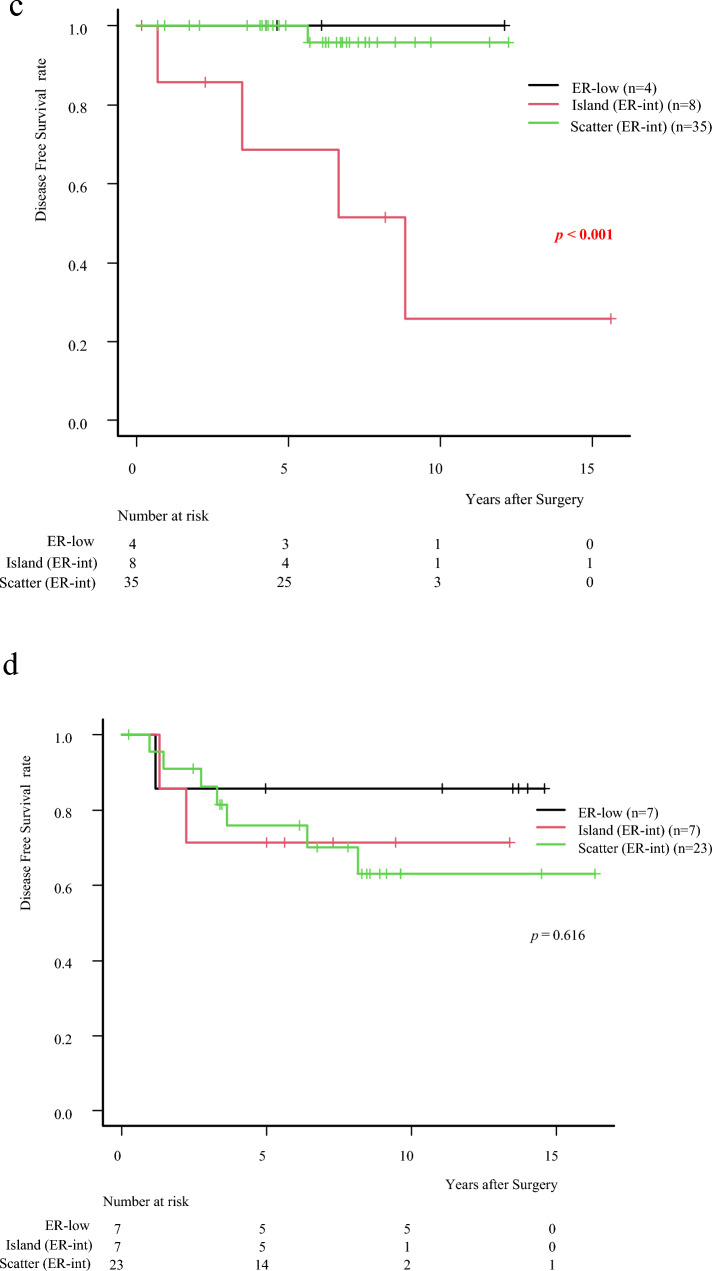
**a** The Kaplan–Meier survival curve illustrates the differences in Disease free survival rates between ER-low and ER-int groups. Ultimately, there was no significant difference in survival between the ER-low and ER-int groups (Log-rank test, *p* = 0.778). The vertical lines in the graph represent censored data, indicating individuals who were lost to follow-up or experienced the event of interest after the last observed time point. **b** Kaplan–Meier survival curve comparing three groups: ER-low (green line), Island (red line), and Scatter (black line). Each line represents the probability of survival over time, with small vertical marks indicating censored data points on each curve. Island group shows a decline in survival over time, while the ER-low and Scatter group exhibits a higher survival rate than the Island group (Log-rank test, *p* = 0.012). **c** The Island type shows significantly worse DFS with endocrine therapy alone (Log-rank test, *p* < 0.0001). **d** The difference is not observed in patients who received both chemotherapy and endocrine therapy (Log-rank test, *p* = 0.6163)

This study examined 104 invasive ductal carcinoma cases, categorizing 23 cases as ER-low (PS2) and 81 cases as ER-int (PS3 and PS4) (Fig. 1) (Table 1). The median age across all patients was 50.5 years, with the PS2 group having a median age of 59 and the PS3,4 group a median age of 50, though this difference was not statistically significant (*p* = 0.0759). No significant differences in tumor size or lymph node metastasis rates were observed between ER-low and ER-int groups, with lymph node metastasis present in 52.9% of patients (*p* = 0.5086). However, the nuclear grade (*p* = 0.0383) and Ki-67 levels (*p* = 0.0006) was significantly higher for the ER-low group. Intensity scores for ER staining and total Progesterone Receptor scores were also significantly lower in the ER-low cases (*p* < 0.001), consistent with prior findings associating ER-low with TN-like characteristics [9–12]. Despite these higher histological factors observed in ER-low patients, no significant differences in prognosis were identified between the ER-low and ER-int groups (*p* = 0.7775) (Fig. 3a).

**Table 1:** Patient characteristics

	All (*n* = 104)		ER-low (*n* = 21)		ER-int (*n* = 83)		ER-low vs ER-int
Age (year), median (IQR)	50.5	(29–77)	59	(34–70)	50	(29–77)	*p* = 0.076
Female sex (%)	104	(100)	23	(100)	81	(100)	
Menopause							*p* = 0.021
Pre- (%)	58	(55.8)	7	(33.3)	51	(61.5)	
Post- (%)	46	(44.2)	14	(66.7)	32	(38.6)	
Invasive size, median (IQR)	20	(4–97)	22	(4–50)	20	(6–97)	*p* = 0.843
T (%)							
T1	53	(51.0)	9	(42.9)	44	(53.0)	
T2	41	(39.4)	10	(47.6)	31	(37.4)	
T3, T4	10	(9.6)	2	(9.5)	8	(9.6)	
LN metastasis (%)							*p* = 0.509
0	49	(47.1)	11	(52.4)	38	(45.8)	
1	18	(17.3)	4	(19.1)	14	(16.9)	
≥ 2	37	(35.6)	6	(28.6)	31	(37.4)	
Nuclear grade*							***p* = 0.038**
1	21	(21.8)	1	(5.9)	20	(25.3)	
2	33	(34.3)	4	(23.5)	29	(36.7)	
3	42	(43.8)	12	(70.6)	30	(38.0)	
Ki-67, median (IQR)	20	(1–90)	50	(2–80)	10	(1–90)	***p* < 0.001**
ER intensity score (%)							***p* < 0.001**
1	36	(34.6)	17	(81.0)	19	(22.9)	
2	57	(54.8)	3	(14.3)	54	(65.1)	
3	11	(10.6)	1	(4.8)	10	(12.1)	
Progesterone receptor, PS (%)							***p* < 0.001**
0–1	44	(42.3)	19	(90.5)	25	(30.1)	
2	9	(8.7)	2	(9.5)	7	(8.4)	
3–4	27	(26.0)	0		27	(32.5)	
5	24	(23.1)	0		24	(28.9)	
HER2 score (%)							*p* = 0.532
0	38	(36.5)	6	(28.6)	32	(38.6)	
1 +	53	(51.0)	13	(61.9)	40	(48.2)	
2 +	13	(12.5)	2	(9.5)	11	(13.3)	
Chemotherapy							***p* < 0.001**
No (%)	47	(45.2)	4	(19.1)	43	(51.8)	
Yes (%)	57	(54.8)	17	(80.9)	40	(62.4)	
Neoadjuvant (%)	19	(18.3)	10	(47.6)	9	(10.8)	
Adjuvant (%)	38	(36.6)	7	(33.3)	31	(37.4)	

This table presents characteristics of all patients (*n* = 104), and a comparison between ER-low (*n* = 21) and ER-int (*n* = 83). Additionally, there is a comparison between ER-low and ER-int. Statistical significance (*p*-values) is provided for each parameter to assess whether there are notable differences between the ER groups

^*^Eight cases without information were excluded

### Prognostic impact of ER-int staining patterns: Island vs. Scatter

To further investigate the impact of ER-int, 84 surgical specimens from patients not receiving neoadjuvant chemotherapy were examined. A total of 73 ER-int samples were classified into two staining patterns: “Island” (*n* = 15) and “Scatter” (*n* = 58) (Fig. 2). The Island group was older (58 vs. 48.5 years, *p* = 0.017) with more postmenopausal patients (66.7% vs. 63.8%, *p* = 0.033). Tumor size, lymph node metastasis, histopathological features, and chemotherapy rate showed no significant differences (Table 2). Kaplan–Meier analysis showed a significant prognostic difference between these patterns (*p* = 0.0116). Patients with the Island pattern, characterized by concentrated and heterogeneous ER expression clusters, exhibited significantly worse DFS compared to both the Scatter and the ER-low group (Fig. 3b). Island patients exhibited rapid DFS decline, particularly within 5 years post-surgery, while ER-low (*n* = 11) and Scatter (*n* = 58) groups maintained stable DFS over time. Island tumors had a marked DFS reduction, indicating a higher recurrence risk. In the endocrine therapy-only group, Island patients had significantly lower DFS than ER-low and Scatter patients (*p* < 0.0001) (Fig. 3c). In contrast, in the chemotherapy group, Island patients showed improved DFS compared to endocrine therapy-only group and there were no significant DFS differences among Island, ER-low, and Scatter patients (*p* = 0.6163) (Fig. 3d).

**Table 2:** Comparison of patient characteristics between the Island and Scatter groups

	Island (*n* = 15)		Scatter (*n* = 58)		Island vs Scatter
Age (year), median (IQR)	58	(41–77)	48.5	(29–76)	***p* = 0.017**
Female sex (%)	15	(100)	58	(100)	
Menopause					***p* = 0.033**
Pre- (%)	5	(33.3)	37	(36.2)	
Post- (%)	10	(66.7)	21	(63.8)	
Invasive size, median (IQR)	27	(9–75)	18	(6–97)	*p* = 0.631
T (%)					
T1	6	(40.0)	36	(62.1)	
T2	8	(53.3)	19	(32.8)	
T3, T4	1	(6.7)	3	(5.2)	
LN metastasis (%)					*p* = 0.255
0	6	(40.0)	32	(55.2)	
1	2	(13.3)	10	(17.2)	
≥ 2	7	(46.7)	16	(27.6)	
Nuclear grade*					*p* = 0.787
1	4	(26.7)	15	(26.3)	
2	5	(33.3)	24	(42.1)	
3	6	(40.0)	18	(31.6)	
Ki-67, median (IQR)	10	(4–30)	10	(1-)	*p* = 0.256
ER intensity score (%)					*p* = 0.096
1	1	(6.7)	13	(22.4)	
2	10	(66.7)	40	(69.0)	
3	4	(26.7)	5	(8.6)	
Progesterone receptor, PS (%)					*p* = 0.277
0–1	5	(33.3)	14	(24.1)	
2	2	(13.3)	4	(6.9	
3–4	7	(46.7)	19	(32.8)	
5	1	(6.7)	21	(36.2)	
HER2 score (%)					*p* = 0.963
0	6	(40.0)	21	(36.2)	
1 +	7	(46.7)	29	(50.0)	
2 +	2	(13.3)	8	(13.8)	
Adjuvant chemotherapy					*p* = 0.623
No (%)	8	(53.3)	35	(60.3)	
Yes (%)	7	(46.7)	23	(39.7)	

This table presents the clinical and pathological characteristics of patients classified into the Island (*n* = 15) and Scatter (*n* = 58) groups. Statistical significance (*p*-values) is provided to determine whether notable differences exist between these two groups

^*^A case without information were excluded

### Prognostic ability of TILs in ER-low and ER-int

**Fig. 4:**
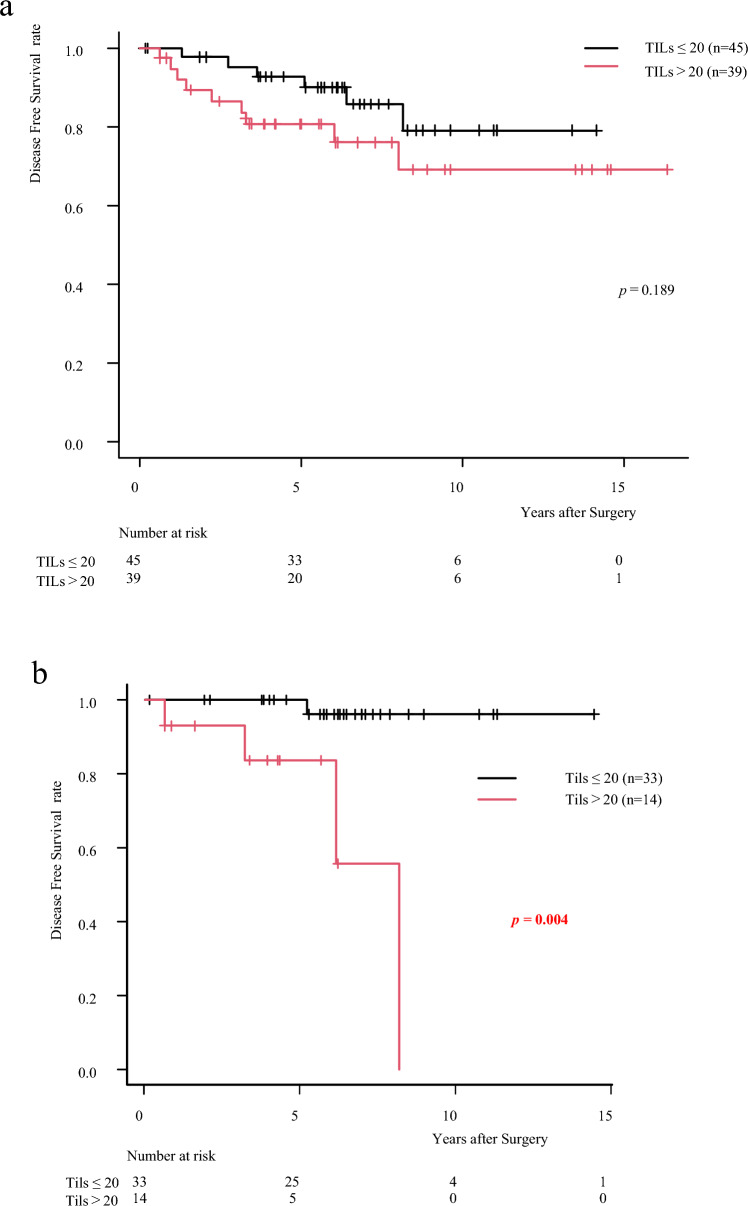

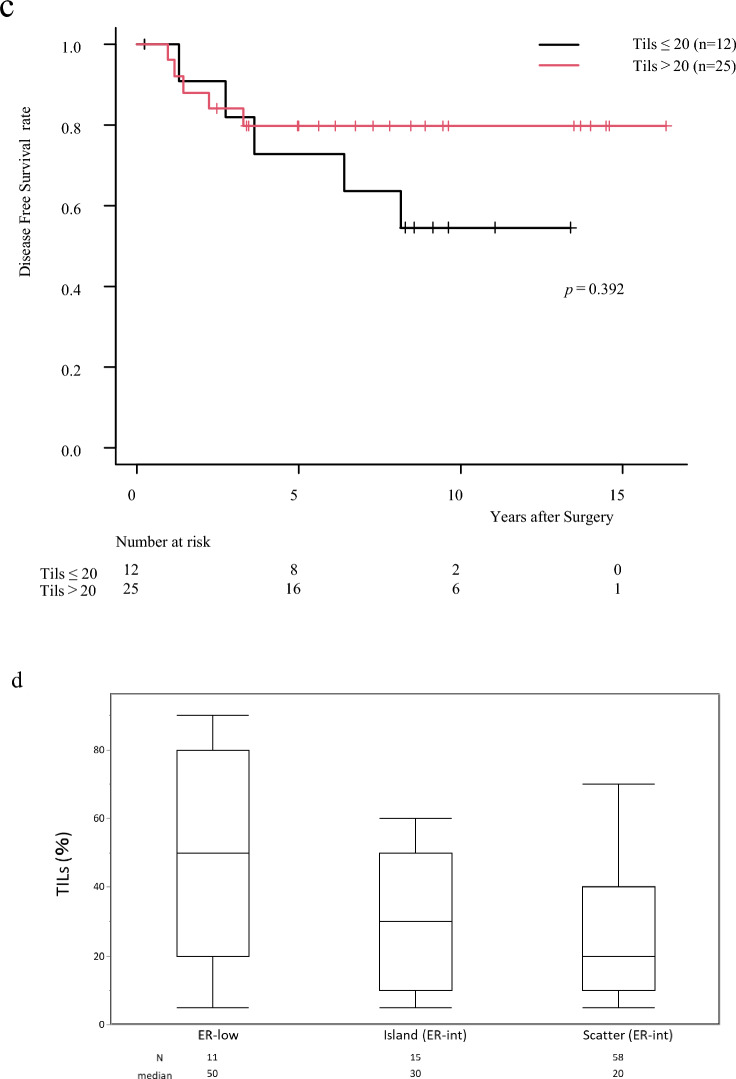
**a** Kaplan–Meier survival curve comparing two groups based on Tils: Tils ≤ 20% (red line) and Tils > 20% (black line). Both curves represent the survival probability over time, with small vertical marks indicating censored data points. **b** Patients with TILs > 20 show significantly worse DFS compared to those with TILs ≤ 20 in the endocrine therapy group (Log-rank test, *p* = 0.004). **c** No significant difference in DFS is observed between TILs > 20 and TILs ≤ 20 in the group receiving both chemotherapy and endocrine therapy (Log-rank test, *p* = 0.392). **d** Boxplot comparing the percentage of TILs across three groups: ER-low, Island ER-int, and Scatter ER-int. This figure indicates that the ER-low group has the highest median TILs, followed by the Island ER-int, with the Scattered ER-int, having the lowest TIL levels

Patients were categorized into two groups based on TIL levels: TILs ≤ 20% (*n* = 45) and TILs > 20% (*n* = 39). Higher TIL levels tended to correlate with worse prognosis, although this trend did not reach statistical significance (*p* = 0.1891) (Fig. 4a). In the endocrine therapy-only group, patients with TILs > 20% had significantly worse DFS than those with TILs ≤ 20% (*p* < 0.0043), showing a marked survival decline within the first five years (Fig. 4b). No significant DFS differences were observed between TILs ≤ 20% and TILs > 20% in the chemotherapy cohort (*p* = 0.3916), with improved outcomes in patients receiving chemotherapy versus endocrine therapy alone (Fig. 4c). These findings suggest that high TIL levels correlate with poor prognosis in the endocrine therapy-only group, while chemotherapy mitigates this negative impact. Additionally, TIL levels varied by ER-int staining pattern: highest in ER-low, followed by Island, and lowest in Scatter (*p* = 0.0721) (Fig. 4d), supporting findings that Island tumors are more sensitive to chemotherapy.

### Transcriptomic and functional insights into ER-int

#### RNA analysis for further understanding of ER-int

**Fig. 5:**
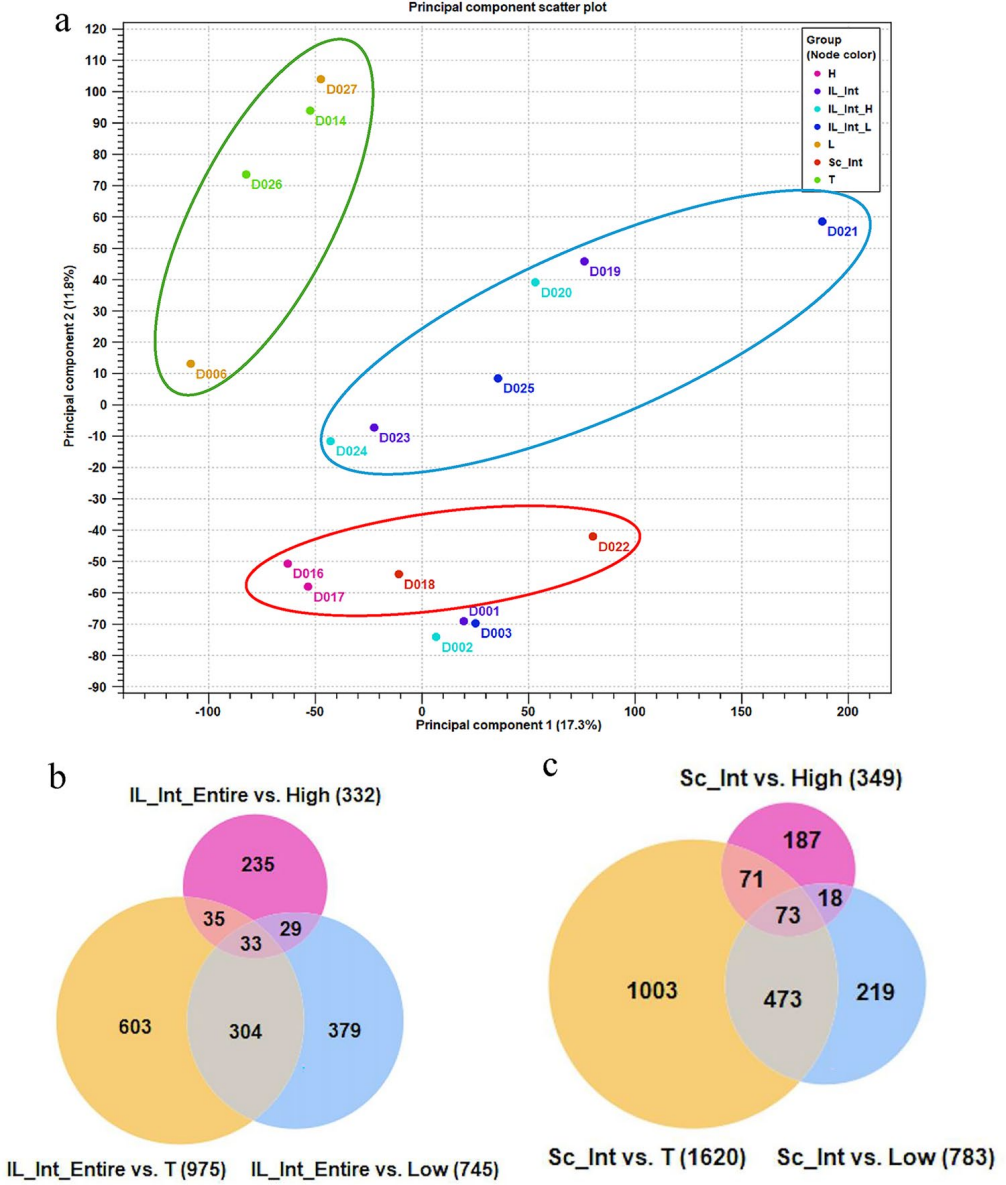
**a** The PCA plots show a clear clustering pattern, with the RNA expression profile distinguishing the L:ER-Low, IL_Int:Island, Sc:Scatter, T:TNBC, and H:ER-high groups The Island subtype samples are further divided by ER High and Low expression regions within the same tumor (IL_int_H:ER high area, IL_int_L:low area). **b** & **c** Common candidates in Island and scatter ER-int tumors. Overlapping candidates in Island and scatter ER-int tumors with FDR 0.05 and fold change 1.5. *IL_int_Entire* Island ER-int tumors, *SC_int* Scatter ER-int tumors, *Hig*: ER-high, *Low* ER-low, *T* TNBC

Principal component analysis demonstrated clustering of ER-low and TN tumors, indicating similar RNA expression patterns. ER-high and Scatter tumors clustered closely, while Island tumors were distributed in between, with some overlap with both high and low expression areas within the same tumor (Fig. 5a, Suppl. Table 2). We further analyzed high and low expression regions within Island tumors to explore intra-tumor heterogeneity. Although differential gene expression analysis identified significantly differentially expressed genes (FDR *q*-value < 0.05, *n* = 2) (see Suppl. Fig. 1), we were unable to establish a functional role for intra-tumor heterogeneity. Therefore, we compared whole Island tumors with Scatter ER-int tumors to examine inter-tumor heterogeneity.

#### Differentially expressed genes (DEGs) between Island and Scatter tumors

**Fig. 6:**
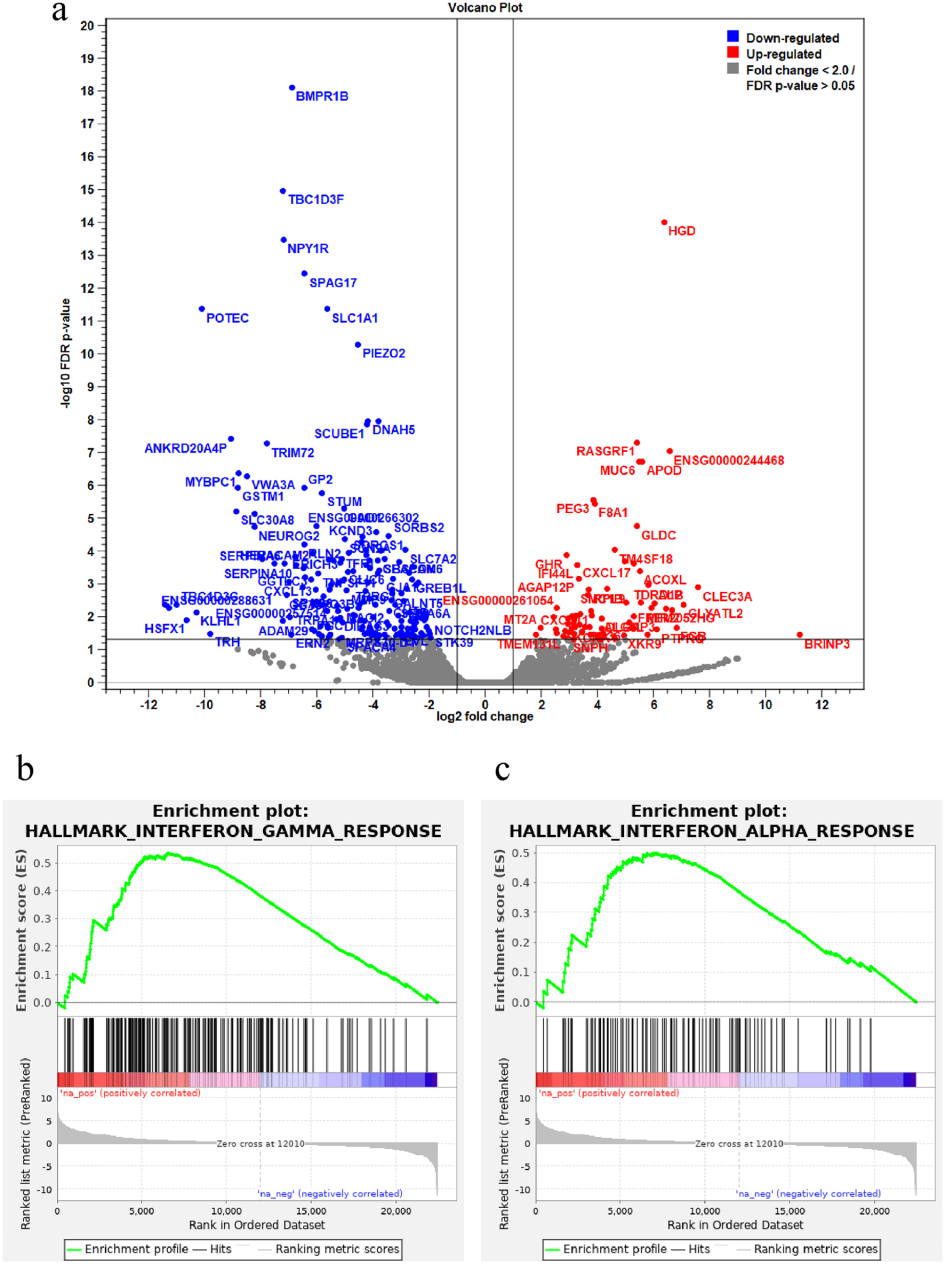

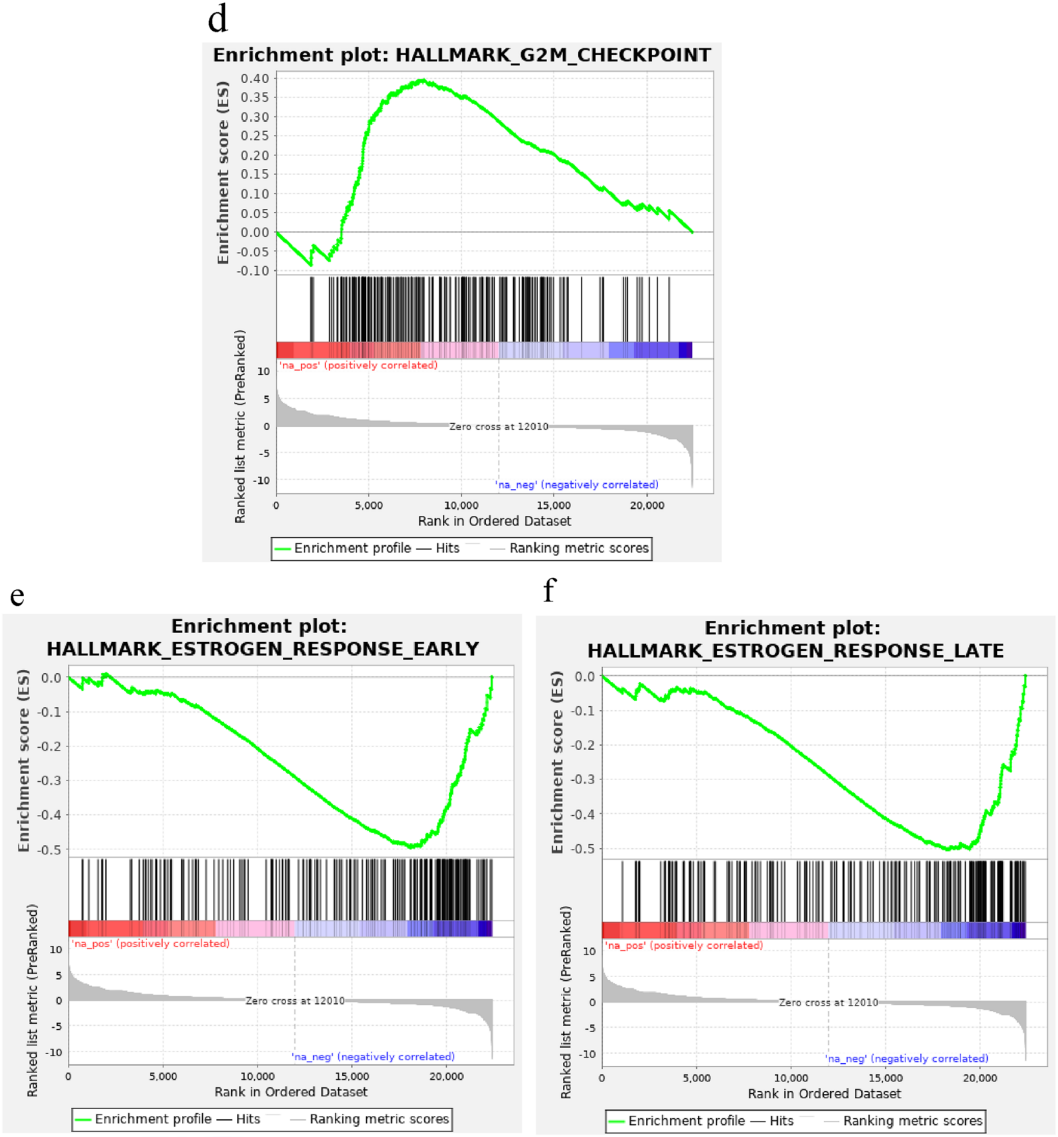
**a** DEGs_Island ER-int tumor vs Scatter ER-int tumor. Volcano plot of differentially expressed genes in Island ER-int group when compared with Scatter ER-int group of tumors. Number of genes that are significantly differentially expressed with *p* ≤ 0.05; FDR *p* value ≤ 0.01 are: Island vs Scatter ER-int: *n* = 138. **b–d** Cancer hallmark enriched gene sets in Island ER-int tumors when compared with Scatter ER-int tumors. **b** Eniched Inteferon gamma response cancer hallmark gene set. **c** Enriched Interferon alpha response cancer hallmark gene set. **d** Enriched G2M checkpoint cancer hallmark gene set with significant positive enrichment scores of nominal p values of 0.0001–001 and FDR *q* values of 0.001, 0.011 and 0.102 respectively. **e**, **f** Cancer hallmark negatively enriched gene sets in Island ER-int tumors when compared with Scatter ER-int tumors. **e** Estrogen response early. **f** Estrogen response late with significant negative enrichment scores of nominal *p* values of 0.0001 and FDR *q* values of 0.0001

Whole transcriptome sequencing revealed that Island and Scatter tumors shared more similar gene expression patterns with ER-high tumors (DEGs with log FC 1.5, *p* < 0.05: *n* = 235 in Island, *n* = 187 in Scatter) than with ER-low (*n* = 379 and 219) and TN (*n* = 603 and 1003) tumors (Fig. 5b). Furthermore, a separate analysis of high and low ER-expressing regions within Island tumors did not demonstrate a strong resemblance between low ER-expressing areas and either ER-low or TN tumor profiles (see Suppl. Fig. 2). The Venn diagram was utilized to identify the common DEGs among the Island and Scatter with ER-high, ER-low and TN subgroups (Fig. 5c), revealing 33 and 73 overlapping DEGs in Island and Scatter, respectively, with significant log FC of 1.5 and FDR *q* < 0.05. Scatter tumors’ DEGs closely mirrored ER-high tumors (see Heatmap, Suppl. Fig. 3). Additional analysis comparing Island and Scatter transcriptomes identified 1546 DEGs with *p* < 0.05 unique to Island tumors. These DEGs appear to influence multiple cancer-related biological processes, potentially contributing to the inter-tumor heterogeneity observed in Island tumors. Further DEG analysis (*p* < 0.05, FDR ≤ 0.01) identified 138 genes significantly differentially expressed in Island versus Scatter tumors (Fig. 6a). To understand the contribution of these DEGs to distinct cancer hallmark enrichment, we conducted gene set enrichment analysis.

#### Gene set enrichment analysis (GSEA) between Island and Scatter tumors

GSEA using preranked genes revealed significant enrichment of interferon-gamma, alpha response, and G2M checkpoint hallmark gene sets in Island tumors compared to Scatter, with positive enrichment scores (nominal *p* < 0.0001–0.001 and FDR *q* < 0.001–0.011) (Fig. 6b–d, Suppl. Table 5). Additionally, downregulation of estrogen response (early and late) hallmark gene sets was observed in Island tumors, with significant negative enrichment scores (nominal *p* < 0.0001, FDR *q* < 0.0001) (Fig. 6e, f, Suppl. Table 6).

#### Functional annotation of ISLANDER genes in Island tumors

Since cancer antigen presentation and immune cell localization are known to play a significant role in TME and heterogeneity, we investigated whether the DEGs identified in Island tumors also contribute to specific pathways. Using DEGs *p* < 0.05 (*n* = 1546) from Island tumors, DAVID and KEGG pathway analysis identified 35 genes contribute to extra cellular matrix (*n* = 7), Gap junction (*n* = 5), PI3 K-AKT signaling (*n* = 10), cyclicAMP signaling (*n* = 7) and focal adhesion genes (*n* = 6) pathways, all significantly upregulated in Island tumors (*p* ≤ 0.05; Suppl. Table 7). After excluding the common DEGs that were found to contribute to the above five pathways, we identified 23 unique “ISLANDER” genes (e.g., ADRB1, AREG, CD36, CFTR, COL2 A1, FREM2, FGF10, GHR, GJA1, LAMB3, LAMC2, and NTRK2) that contribute to these pathways’ upregulation in Island tumors, potentially driving inter-tumor heterogeneity relative to Scatter tumors.

#### Role of ISLANDER genes in prognosis of ER-int

**Fig. 7:**
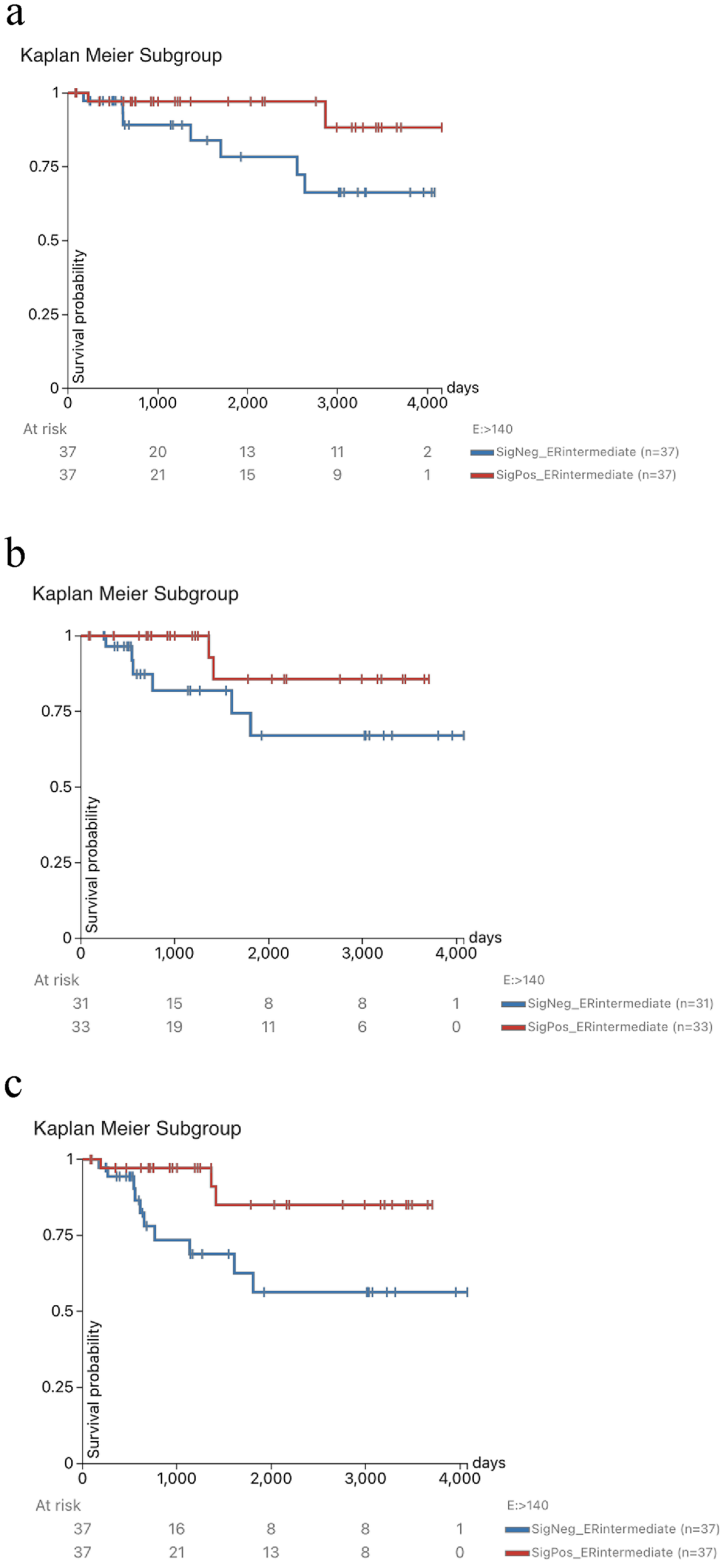
**a**–**c** Prognostic ability of 23 ISLANDER genes in TCGA BRCA ER-int subgroup. **a** Disease specific survival with *p* value of 0.0825. **b** Disease free interval with *p* value of 0.0915. **c** Progression free interval with *p* value of 0.00182. With 23 gene expression median (E) of 140.0

To examine ISLANDER genes (*n* = 23), we hypothesized that an Island-specific gene signature could classify ER-int group into high and low expression subgroups with prognostic ability. After excluding the normal, metastatic and tumors with no expression data from the TCGA BRCA cohort, and with gene expression median of 140.0 we found that the 23 ISLANDER genes divided ER-int tumors into high and low expression subgroups. These subgroups demonstrated significant DSS, DFI and PFI, with p-values of 0.0825, 0.0915, 0.0182 and log rank test statistics of 3.014, 2.846 and 5.570 respectively (Fig. 7a–c).

Overall, these results suggest the possibility of Island tumors to be heterogeneous to Scatter tumors, with distinct level of TILs, cancer hallmarks, immunogenicity, extra cellular matrix and other significant pathways.

## Discussion

The findings of this research provide new insights into understanding the complexity of ER-positive breast cancer, especially in populations where ER expression is different with in the tissue and tumor subtypes. The aim of the current study is to investigate heterogeneity between Island and Scatter ER-int tumors. Using TIL levels, we investigated the prognostic ability of TILs, and RNA sequencing data to report potential enriched genes and pathways contributing to the inter-tumor heterogeneity. To our knowledge, this is the first study to report inter-tumor heterogeneity between Island and Scattered components of the same tumors.

When comparing ER-low (PS2) breast cancers to those with ER-int (PS3, PS4), we observed significant differences in nuclear grade, Ki-67 index, and total progesterone receptor scores, supporting the notion that ER-low exhibit behavior like TN breast cancer. Specifically, ER-low were associated with higher nuclear grades and Ki-67 levels, indicating more aggressive tumor behavior. These results are consistent with previous reports [9–12] suggesting that ER-low shares pathological features with TN breast cancer. Despite these unfavorable pathological characteristics, no significant difference in DFS was observed between the ER-low and ER-int groups, highlighting the need for further investigation into the ER-int group. Unlike ER-low, ER-int is often treated in the same way as ER-high positive, i.e., endocrine therapy is often the treatment of choice. We hypothesized that, alternative treatment indicators should be sought and treatment strategies for this subgroup.

A key finding of this study is the identification of two distinct ER staining patterns—Island and Scatter—in patients with ER-int expression. The patients with Island staining, characterized by heterogeneous clusters of ER-positive cells, demonstrated significantly worse DFS compared to those with the Scatter or ER-low. This suggests that even within the same ER category, differences in intertumoral ER distribution may serve as important prognostic indicators. Particularly in the group receiving endocrine therapy alone, the worse prognosis of the Island suggests that this subgroup may have weaker responses to endocrine therapy and may require additional treatments such as chemotherapy. In recent years, cases of HER2 low expression (HER2-low, IHC 1 + or 2 +/ISH negative) have been identified among those diagnosed with ER-positive HER2-negative breast cancer, and they are attracting attention as a target for treatment with anti-HER2 drugs [33]. As we have seen, HER2 expression in ER-positive breast cancer is not negligible, but in this study, there was no difference in HER2 status between Island and Scatter tumors. Therefore, it was thought that the prognosis differences between these tumor types were not affected by HER2 expression.

The assessment of TILs, which predominantly include T cells, B cells, and macrophages infiltrating tumor tissues, offers insight into immune responses against tumors and play a critical role in modulating the immune response within tumors [23]. In TN breast cancer, high TIL levels are generally associated with a favorable response to chemotherapy and better prognosis [34–36]. However, the role of TILs in ER-positive breast cancer remains unclear [37, 38]. In this study, TILs were evaluated to examine the need for chemotherapy for ER-low and ER-int. High TIL levels were associated with poorer prognosis, especially within five years post-surgery, in patients who received endocrine therapy alone. However, in patients who received chemotherapy, this adverse effect was mitigated. This suggests that even in ER-low and ER-int breast cancer, high TIL levels are an indicator of a good response to chemotherapy and a poor prognostic factor in the absence of chemotherapy. Interestingly, TILs were most abundant in the ER-low group, followed by the Island, with the fewest in the Scatter type. This distribution aligns with the aggressive features observed in the ER-low and Island groups, suggesting that high TIL levels may be related to an increased immune response against these aggressive subtypes. However, in the absence of chemotherapy, this immune response alone may not be sufficient to improve prognosis.

Island ER-int transcriptome exhibited a reduced estrogen response and enriched immune components like interferon alpha and gamma cancer hallmark gene sets when compared to the Scatter ER-int. One of the recent studies [13] reported that both ER-low and ER-int could mimic immune landscape that of TN. In our study, we found no evidence that the transcriptome of low ER-expressing regions in the island strongly resembles the TN or ER-low expression regions. On The other hand, we identified a complex interplay involving CD36, CXCL11, GZMB, MT2 A, and CD8 + T cells that regulates immune function and tumor cell cycle processes. The upregulated expression of these genes in Island tumors contributes to inter-tumor heterogeneity by modulating immune evasion mechanisms and cell cycle checkpoints, distinguishing them from Scatter tumors. CD36 is known to effect CD8 + T cells via ferroptosis and lipid metabolism [39, 40]. GZMB is another molecule that we found to be significantly expressed in Island tumors, is a known cytotoxic factor that plays a significant role in the TME and as predictor of immune check point blockade benefits [41]. Along with these molecules, we also found 23 ISLAND genes that significantly contribute to the enrichment of genes that could influence the extra cellular matrix, GAP junctions and focal adhesions leading to differential cancer and stromal cell interactions, tumor immune microenvironment and tumor growth. To determine the endpoints of DSS, DFI, and PFI, Liu et al., censored patients who remained disease-free and died from unrelated causes. Doing so, this allows us to assume that the TCGA pan-cancer cohort patients could have eventually experienced end to life due to the indexed cancer [32]. However, although the 23 Island genes in this study showed significant PFI in the TCGA BRCA cohort, the lack of treatment modality information among the ER-positive breast cancers in this cohort warrants caution in interpreting the PFI significance and use as an end-point.

The results of this study provide important implications for the treatment of breast cancer patients with ER-int expression. Following this study, we propose Island subtype as a novel breast cancer subtype that needs different treatment strategies. Our preliminary observation of intra-tumor heterogeneity in ER staining patterns, TIL levels and transcriptome suggest that more refined treatment approaches are needed to validate our findings to classify Island as a distinct subgroup.

Patients with detected Island staining were shown to have a poor prognosis with endocrine therapy alone, in contrast to patients with the same proportion of ER-positive cells but with detected Scatter staining, suggesting the need for improved combination therapy including chemotherapy and immunotherapy. Molecular profiling may help further distinguish and stratify ER-int subgroups to guide personalized treatment strategies.

## Limitations and future directions

The relatively small sample size within the subgroups may affect the generalizability of these results. Additionally, the exclusion of patients who received neoadjuvant chemotherapy may have introduced selection bias. Regarding staining classification, only cases with clear intra-tumor heterogeneity were defined as Island, while ambiguous cases may have been classified as Scatter, highlighting the need for standardized criteria. In this study, we found strong correlation with certain immune molecules and cancer development pathways. However, since correlation cannot always lead to causation, we are carrying out single cell and whole exome analysis in Island and Scatter tumors to understand the TME and cell composition to establish the causation of heterogeneity in Island tumors. Future studies should aim to validate these findings in larger prospective cohorts and further investigate the molecular characteristics of the island and Scatter tumors to understand the biological factors driving differences in prognosis.

## Conclusion

In conclusion, this study suggests that ER staining patterns, TILs and transcriptome may serve as important prognostic markers to identify Island groups. Our findings support that Island tumors to have heterogeneous immunogenicity, and TME associated with poor prognosis, suggesting endocrine therapy alone may be insufficient. These findings underscore the need for further investigation of island ER-int tumors to establish the island tumor as a distinct breast cancer subtype.

## Supplementary Information

Below is the link to the electronic supplementary material.

Supplementary file1 (PPTX 432 KB)

Supplementary file2 (XLSX 4550 KB)

## Data Availability

The patient-level clinical data generated and analyzed during this study are not publicly available due to patient privacy concerns, but are available from the corresponding author on reasonable request. The RNA expression data that support the findings of this study are included in the Supplementary Information.

## Abbreviations

ER: Estrogen receptor
TN: Triple negative
TME: Tumor microenvironment
IHC: Immunohistochemistry
PS: Proportion score
DFS: Disease free survival
TILs: Tumor-infiltrating lymphocytes
FFPE: Formalin-fixed, paraffin-embedded
DEG: Differentially expressed gene
NES: Normalized enrichment score
FC: Fold change
DAVID: Database for Annotation, Visualization, and Integrated Discovery
KEGG: Kyoto Encyclopedia of Genes and Genomes
TCGA: The Cancer Genome Atlas
