# Correction to: Prognostic impact of ER‑staining patterns and heterogeneity of ER positive HER2 negative breast cancer

**DOI:** 10.1007/s12282-025-01729-z

**Published:** 2025-06-09

**Authors:** Yumiko Akita, Ravi Velaga, Madoka Iwase, Satoko Shimada, Toyone Kikumori, Dai Takeuchi, Yuko Takano, Takahiro Ichikawa, Tomoki Ebata, Norikazu Masuda

**Affiliations:** 1 Department of Breast and Endocrine Surgery, Nagoya University Graduate School of Medicine, 65 Tsurumai-cho, Showa-ku, Nagoya, Aichi 466-8550 Japan; 2 Department of Pathology, Nagoya University Hospital, 65 Tsurumai-cho, Showa-ku, Nagoya, Aichi 466-8550 Japan; 3 Department of Clinical Oncology and Chemotherapy, Nagoya University Hospital, 65 Tsurumai-cho, Showa-ku, Nagoya, Aichi 466-8550 Japan; 4 Division of Surgical Oncology, Department of Surgery, Nagoya University Graduate School of Medicine, 65 Tsurumai-cho, Showa-ku, Nagoya, Aichi 466-8550 Japan; 5 Department of Breast Surgery, Graduate School of Medicine, Kyoto University, 54 Shogoin Kawahara-cho, Sakyo-ku, Kyoto, 606-8507 Japan

**Correction to: Breast Cancer** 10.1007/s12282-025-01716-4

In this article the title was incorrectly given as ‘Prognostici of ER-staining patterns and heterogeneity of ER positive HER2 negative breast cancer’ but should have been ‘Prognostic Impact of ER‑staining patterns and heterogeneity of ER positive HER2 negative breast cancer’. The original article has been corrected.

