# Supplementary material for: Prognostic impact of ER-staining patterns and heterogeneity of ER positive HER2 negative breast cancer
Source: Breast Cancer. 2025 May 18;32(5):917–34. doi: 10.1007/s12282-025-01716-4 (PMC12394359; doi:10.1007/s12282-025-01716-4)
Supplement: Supplementary file 1 — Supplementary file1 (PPTX 432 KB) [file 12282_2025_1716_MOESM1_ESM.pptx]

## Slide 1
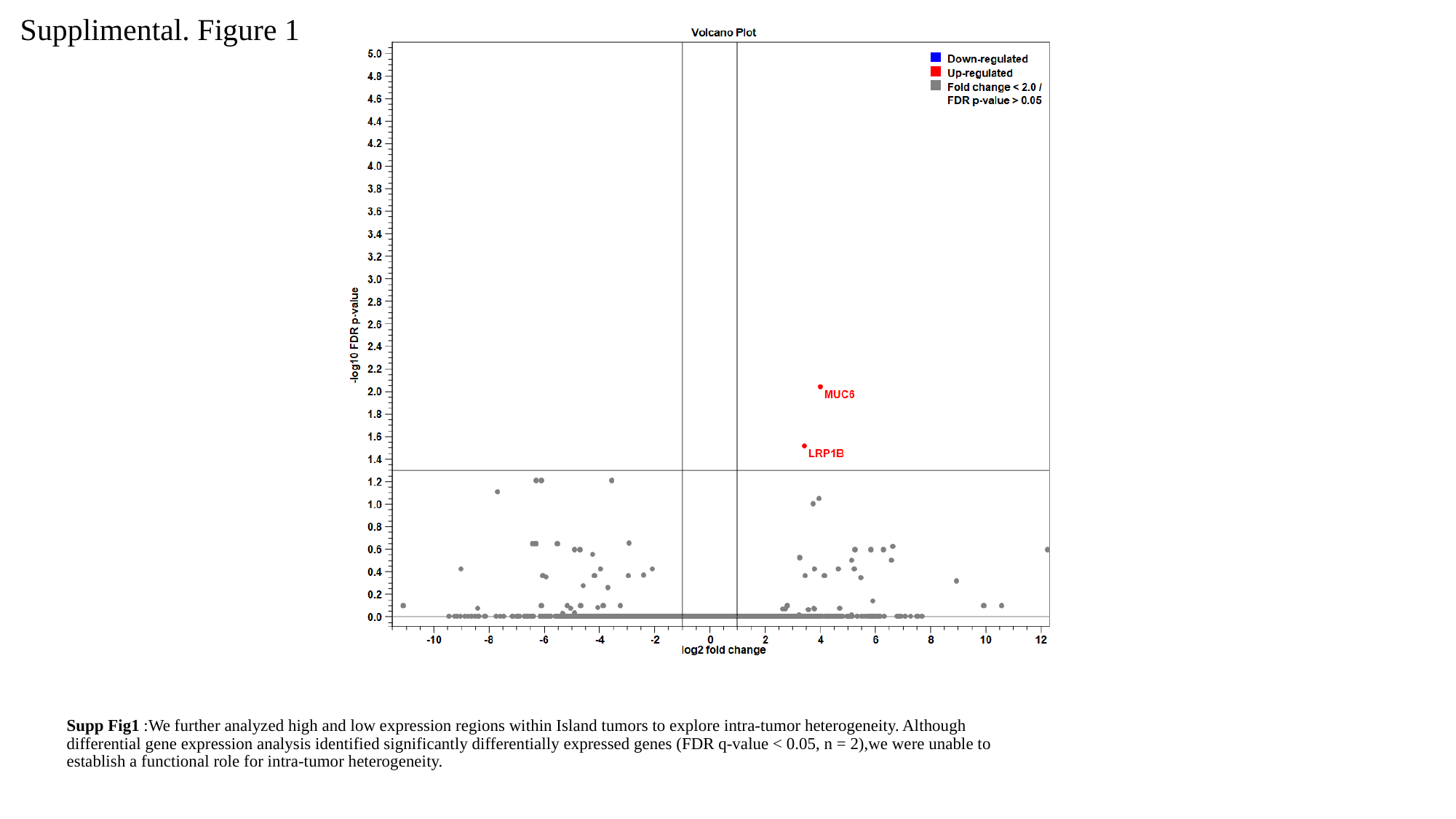

Supplimental. Figure 1
# Supp Fig1 :We further analyzed high and low expression regions within Island tumors to explore intra-tumor heterogeneity. Although differential gene expression analysis identified significantly differentially expressed genes (FDR q-value < 0.05, n = 2),we were unable to establish a functional role for intra-tumor heterogeneity.

## Slide 2
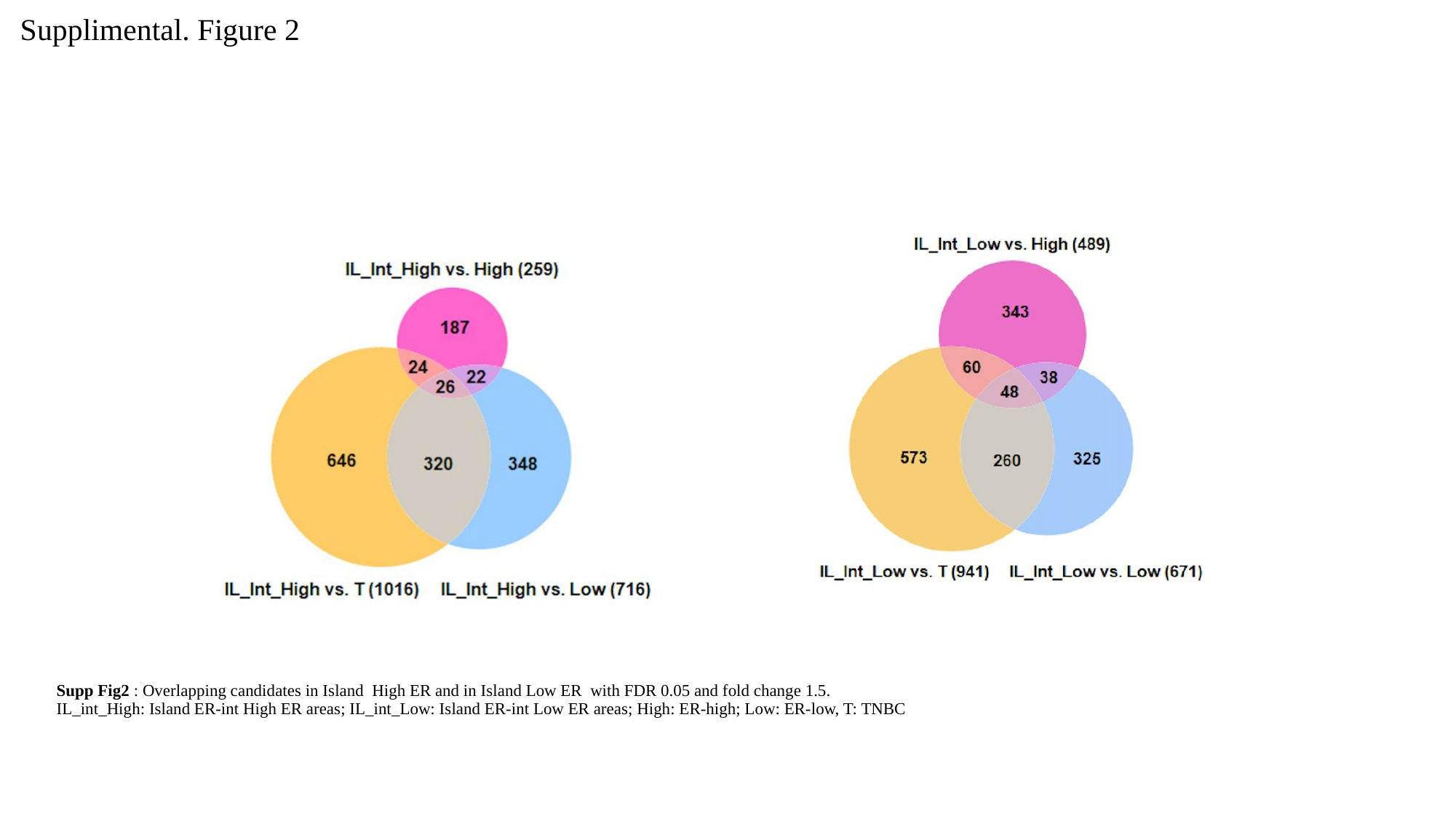

Supplimental. Figure 2
# Supp Fig2 : Overlapping candidates in Island High ER and in Island Low ER with FDR 0.05 and fold change 1.5. IL_int_High: Island ER-int High ER areas; IL_int_Low: Island ER-int Low ER areas; High: ER-high; Low: ER-low, T: TNBC

## Slide 3
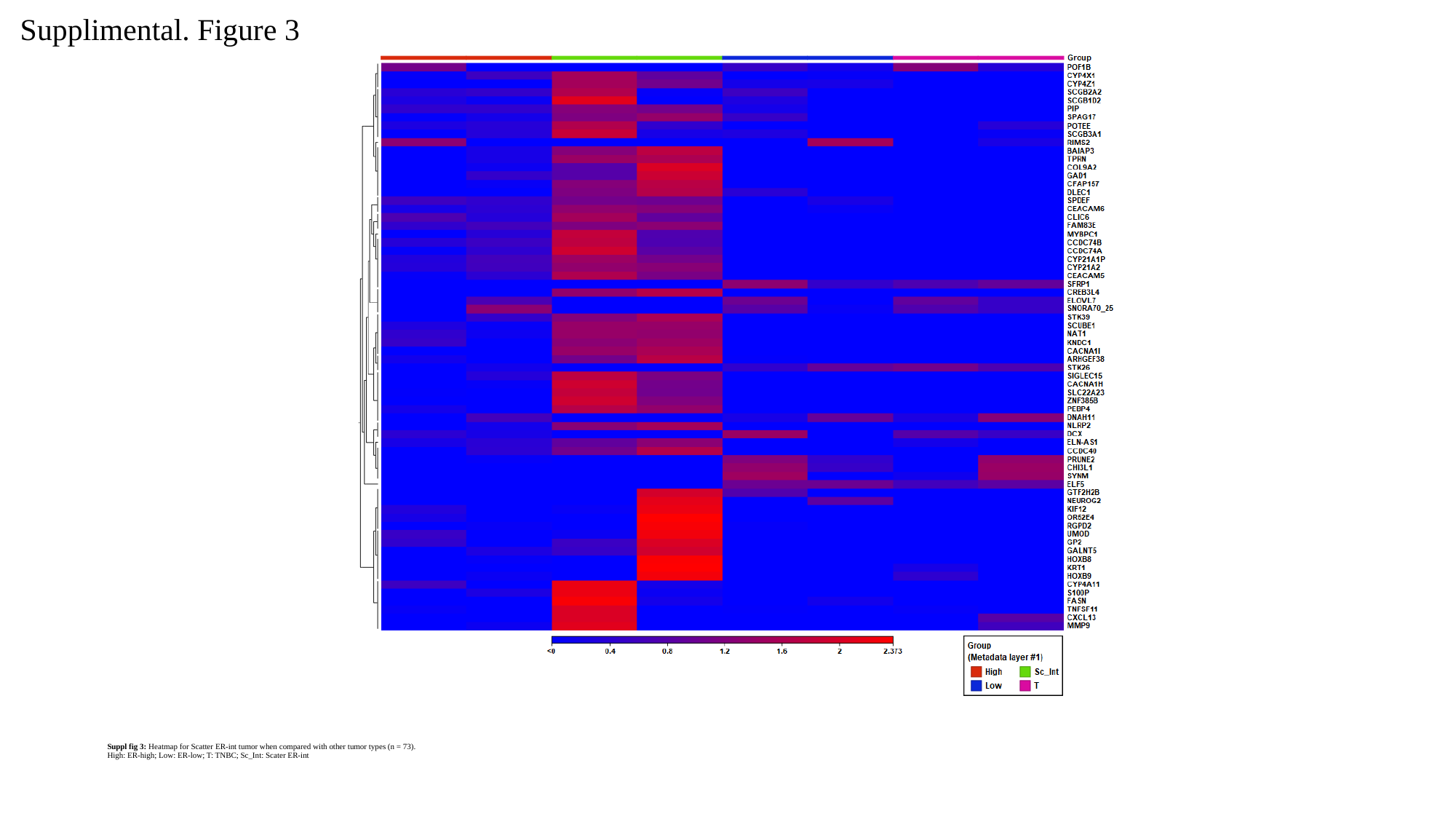

Supplimental. Figure 3
# Suppl fig 3: Heatmap for Scatter ER-int tumor when compared with other tumor types (n = 73). High: ER-high; Low: ER-low; T: TNBC; Sc_Int: Scater ER-int

## Slide 4
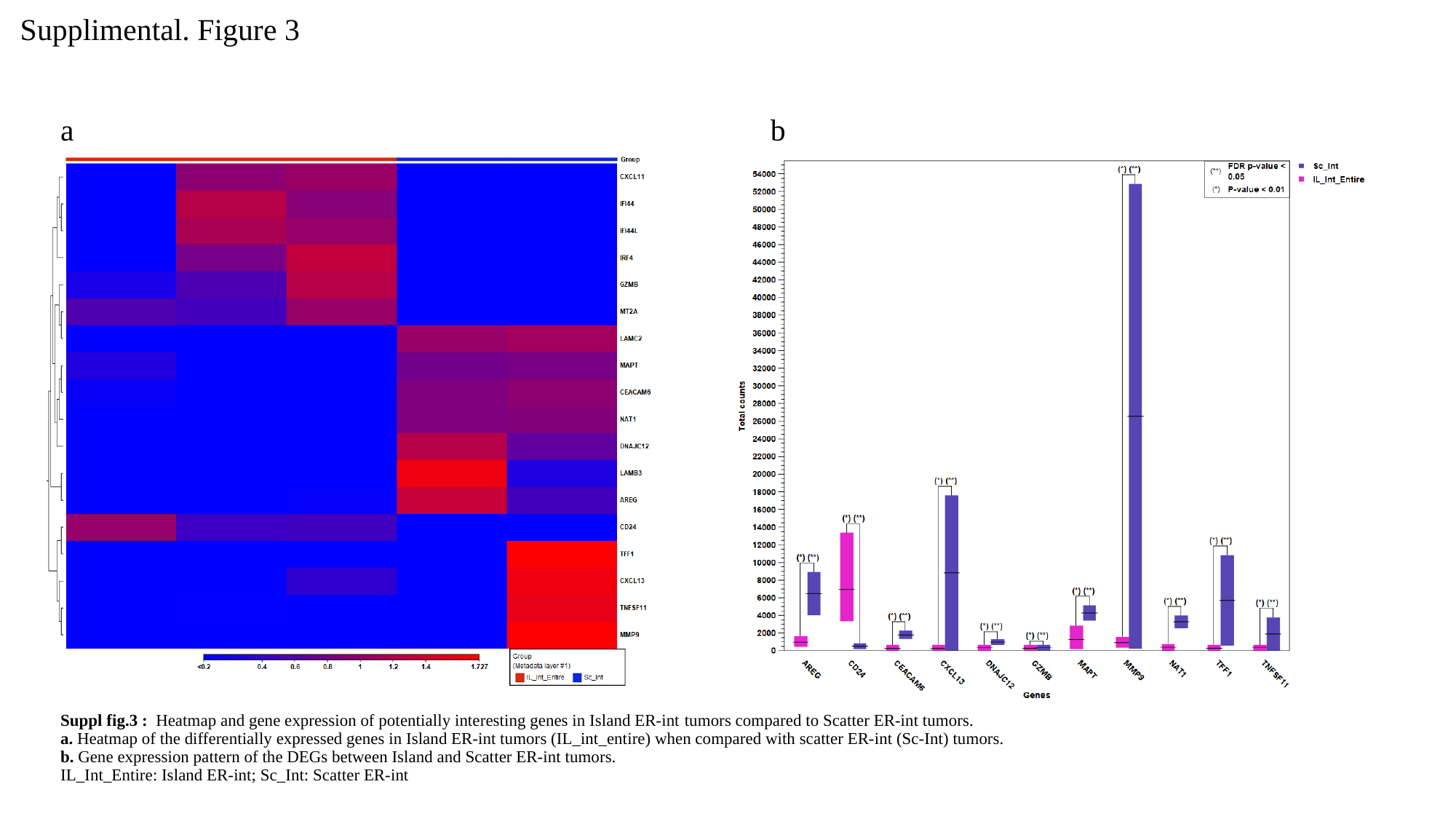

Supplimental. Figure 3
a
b
Suppl fig.3 : Heatmap and gene expression of potentially interesting genes in Island ER-int tumors compared to Scatter ER-int tumors.
a. Heatmap of the differentially expressed genes in Island ER-int tumors (IL_int_entire) when compared with scatter ER-int (Sc-Int) tumors.
b. Gene expression pattern of the DEGs between Island and Scatter ER-int tumors.
IL_Int_Entire: Island ER-int; Sc_Int: Scatter ER-int
